# Annotations

**Published:** 1906-03-24

**Authors:** 


### ANNOTATIONS.

### Borax and Boric Acid as Food Preservatives.

The attempt to determine whether borax and boric acid can be used as food preservatives without prejudice to health has led to much conflict of opinion, and the latest effort in this direction is not likely to escape the fate of its predecessors. To avoid the objection that what may be true of the action of these substances on the lower animals is not necessarily true in regard to human beings, Dr. H. W. Wiley, of the Board of Agriculture of the United States Government, has carried out a series of experiments on human beings. The tests included 30 individuals, and were extended over many months. The results were far from alarming, either in character or degree, but Dr. Wiley states that under the use of the preservatives there was a slight tendency to a decrease of the body-weight, with a diminished excretion of nitrogen and an increased elimination of phosphorus. Other observations not susceptible of numerical calculation included some disturbance of appetite, of digestion, and of the general health. But since the publication of these conclusions the whole conditions of the experiments have been subjected to a searching scrutiny by Dr. Oscar Liebreich, who challenges the findings in no uncertain terms. The average loss of weight, Dr. Liebreich contends, was so slight that it might well be due to chance occurrences at the preservative table, and even if due to the preservative does not necessarily prove this to be an injurious agent. In further support of his criticisms Dr. Liebreich draws particular attention to the statement of the report declaring that "at the end of the year, after the final 'after period' had been passed," the subjects of the experiment "appeared to be, and declared themselves to be, in better physical condition than when they entered upon the experimental work seven months before." Altogether it will have to be admitted that the serious charges brought against the food preservatives in question in certain quarters are not proved to be true by this latest series of experiments.

### The Care of Children in the Colonies.

"The ultimate test of the prosperity and stability of a new colony lies in the health of the children born and reared in it." So runs the preface to a small hand-book from the pen of Dr. Lilian Austin Robinson. The truth expressed in the opening sentence of the preface quoted above sufficiently justifies the appearance of a book which we have no hesitation in recommending as an excellent specimen of its kind. The writer calls it "a book for mothers" and dedicates it to her children. The dedication establishes the author's apprenticeship in one of the best schools of pædiatrics, that, namely, of motherhood. To this immense advantage Dr. Robinson has added a sound knowledge of the underlying principles of the hygiene and therapeusis of childhood, both mental and bodily, with the result that she has produced a book accurate enough, popular enough, and sympathetic enough to command a place in the library of all those of our countrywomen who are compelled by force of circumstances to be on occasions their own family doctor. This comprehensive blessing does not imply that we are in absolute agreement with everything advocated by the author, but the points of divergence concern minor matters. For instance, under the heading "croup" the author retains the term "membranous croup" as a disease distinct from diphtheria. Now we are prepared to admit that other organisms besides the Klebs-Löffler bacillus are capable of producing a membranous laryngitis, but they do it so seldom that in our opinion a popular hand-book is better without a distinction which has often in the past proved very pernicious. Again, in the treatment of croup by the old routine method—namely, a drachm dose of ipecacuanha wine, we are told that "should it fail to act (as an emetic) within the first ten minutes vomiting must be induced . . . as the drug is a depressing one if allowed to enter the system in such a dose." This is the old teaching, but bad practice. There will, we fancy, be many who share with us the opinion, both that a drachm dose of ipecacuanha wine rarely succeeds as an emetic, and that many drachms can be retained without untoward results. But such small details do not detract appreciably from the merits of a thoughtful and well-written piece of work.

### The Housing and Feeding of Chinese Coolies.

The political issues of the Chinese labour question in South Africa do not concern us; but the housing and feeding of the Chinese coolies on British territory is, of course, a matter of great interest, and we have read the report which the medical officer of Johannesburg has prepared with much satisfaction. Seven mines were visited by this official without notice to the mine managers. In accordance with the recommendations of the Transvaal Medical Service, the Chinese are housed in substantial buildings, containing clean and comfortable rooms, some of them being quite gay in appearance. The atmosphere of the rooms, even with the full complement of persons present, is described as invariably free from the "muggy" smell so generally noticeable in lodging-houses and barrack-rooms in England. An ample sufficiency of baths, hot and cold, and of latrine accommodation is provided. As to the diet, the daily menu includes plenty of rice, fresh meat or fish, white bread, tea, salt, and a good variety of fresh vegetables. When the men are indisposed, they receive competent medical attendance in well-equipped special hospitals, and at one of these there is a resident qualified Chinese doctor, in addition to the English visiting staff. In conclusion, the author of the report, who has had ten years' experience of the housing of the labouring classes as medical officer of health for the county of Shropshire, the borough of Stockport, and East Kent, states that the conditions under which the Chinese live in the compounds within the municipality of Johannesburg are "very much superior as regards housing and food to those which the unskilled labourer can command in either the manufacturing or rural districts of England."

